# Comparison of microbial profiles between hospital wastewater and river water

**DOI:** 10.1007/s00438-026-02478-0

**Published:** 2026-06-29

**Authors:** Lazaros A. Gagaletsios, Tsolaire Sourenian, Dimitrios Karpouzas, Ibrahim Bitar, Costas Papagiannitsis

**Affiliations:** 1 Department of Microbiology, Faculty of Medicine, University of Thessaly, Larissa, Greece; 2 Department of Microbiology, Faculty of Medicine and University Hospital in Plzen, Charles University, Plzen, Czech Republic; 3 Faculty of Biochemistry & Biotechnology, University of Thessaly, Larissa, Greece; 4 Department of Microbiology, University Hospital of Larissa, Larissa, Greece

**Keywords:** Hospital wastewater, River water, Enterobacterales, Pseudomonadales, Aeromonadales, Carbapenemases, Multidrug-resistant bacteria

## Abstract

This study aimed to compare the microbial profiles between hospital wastewater and river water to assess the dissemination of clinical isolates into the environment. Two types of water samples were collected from sampling sites which were geographically close (wastewater from the University Hospital of Larissa and river water from the Pineios River). Gram-negative bacteria isolated from both sample types were identified using MALDI-TOF. Furthermore, the minimum inhibitory concentration (MIC) of antibiotics were evaluated. A total of 54 Gram-negative isolates, belonging to diverse species, were collected from wastewater sample and river sample. All isolates were classified as MDR, exhibiting resistance to at least one agent from more than three different antibiotic classes. Based on species identification and susceptibility profiles, 27 isolates (19 from wastewater and 8 from river-water) were selected to be further characterized by whole-genome sequencing (WGS). Analysis of WGS data, revealed the presence of different STs, even in isolates belonging to the same bacterial species. Additionally, WGS data showed that carbapenemase-encoding genes were identified in the majority of isolates. PlasmidFinder identified a huge variety of plasmid replicons among the isolates studied. In conclusion, both hospital wastewater and river water contained isolates carrying clinically relevant resistance determinants, such as carbapenemase-encoding genes. The presence of these pathogenic bacteria in the river poses a significant public health concern. Although we could not identify the origin of MDR bacteria in the river sample, these findings highlight the growing threat of antimicrobial resistance in the environment and underscore the urgent need for improved treatment methods and stricter surveillance to control its spread.

**Supplementary Information:**

The online version contains supplementary material available at 10.1007/s00438-026-02478-0.

## Introduction

The role of antibiotics and antibiotic resistance in environmental contaminants has been largely understudied, unlike their long-recognized impact in clinical settings (Xu et al. 2026; Gao et al., 2022). Consequently, the growing prevalence of multidrug-resistant organisms presents a critical challenge for contemporary healthcare (Ranibar and Alam 2023).

Wastewater from hospitals can be hazardous, as it often contains radioactive, chemical, and pharmaceutical waste along with disease-causing microorganisms, threatening both public health and the environment (Verlicchi et el. 2010). The overuse of antibiotics in humans and livestock accelerates the development of antibiotic resistance, leading to resistance genes appearing in environments like hospital wastewater (Iversen et al. 2002). Research confirms that this wastewater serves as a breeding ground for resistant bacteria, substantially increasing the amount released into the natural environment (Patnaik et al. 2025).

Furthermore, freshwaters, like rivers, are increasingly recognized as reservoirs for antimicrobial resistance since they are subjected to urban and agricultural runoff. The widespread use of antibiotics in livestock and agriculture has been linked to the emergence of antibiotic-resistant bacteria and the dissemination of antibiotic resistance genes (Cufaoglu et al. 2021). Also, rivers serve as hotspots for the development and dissemination of metal compounds and metal resistance (Gupta et al. 2023). Metals influence the composition and diversity of bacterial community by affecting microbial enzyme activity, metabolic diversity, and the utilization of organic substrates (Du et al. 2018). In addition, heavy metals, even at sub-lethal concentrations, can promote antibiotic resistance through horizontal gene transfer (Seiler and Berendonk 2012). Thus, rivers are critical points for monitoring the presence and distribution of antimicrobial resistance determinants in the environment.

The principal public health threat is the potential horizontal transfer of antibiotic resistance genes from environmental bacteria to human pathogens (Kruse et al.,^1999^). This risk is critically amplified by hospital wastewater, which introduces both substantial loads of resistant bacteria and bioactive antibiotic residues into municipal sewers. These residues exert direct selection pressure, suppressing susceptible microorganisms and thereby promoting the proliferation of resistant strains (Pinamonti et al. 2025). Consequently, hospital waste effluent increases the numbers of resistant bacteria in the recipient sewers by both mechanisms of introduction and selection for resistant bacteria (Al-Ahmand et al., 1999).

In addition, Wastewater Treatment Plant (WWTP) were initially built to lower oxygen demand, nitrogen, phosphorus pollution and total suspended solids (TSS), while eliminating disease-causing microbes was a lower priority (Lucas et al., 2014). However, a wide range of both known and unknown pathogens, including bacteria, are a significant concern in wastewater. For instance, fecal matter can contain up to 10^6^ pathogenic bacteria per gram (Kokkinos et al. 2015). The most common gram-negative pathogenic bacterium in wastewater is *Salmonella* (Espigares et al. 2006). Although microbes can strongly influence effluent quality, the full variety of wastewater microbiota is not yet well studied (Wang et al., 2014). Thus, the aim of the current study was to examine presence of the MDR bacteria in a river water sample and a hospital wastewater sample and, then, subsequently to evaluate if there is a cross-contamination between hospital wastewater and river water, based on WGS characteristics of the isolated bacteria.

## Materials and methods

### Sample collection

**Fig. 1:**
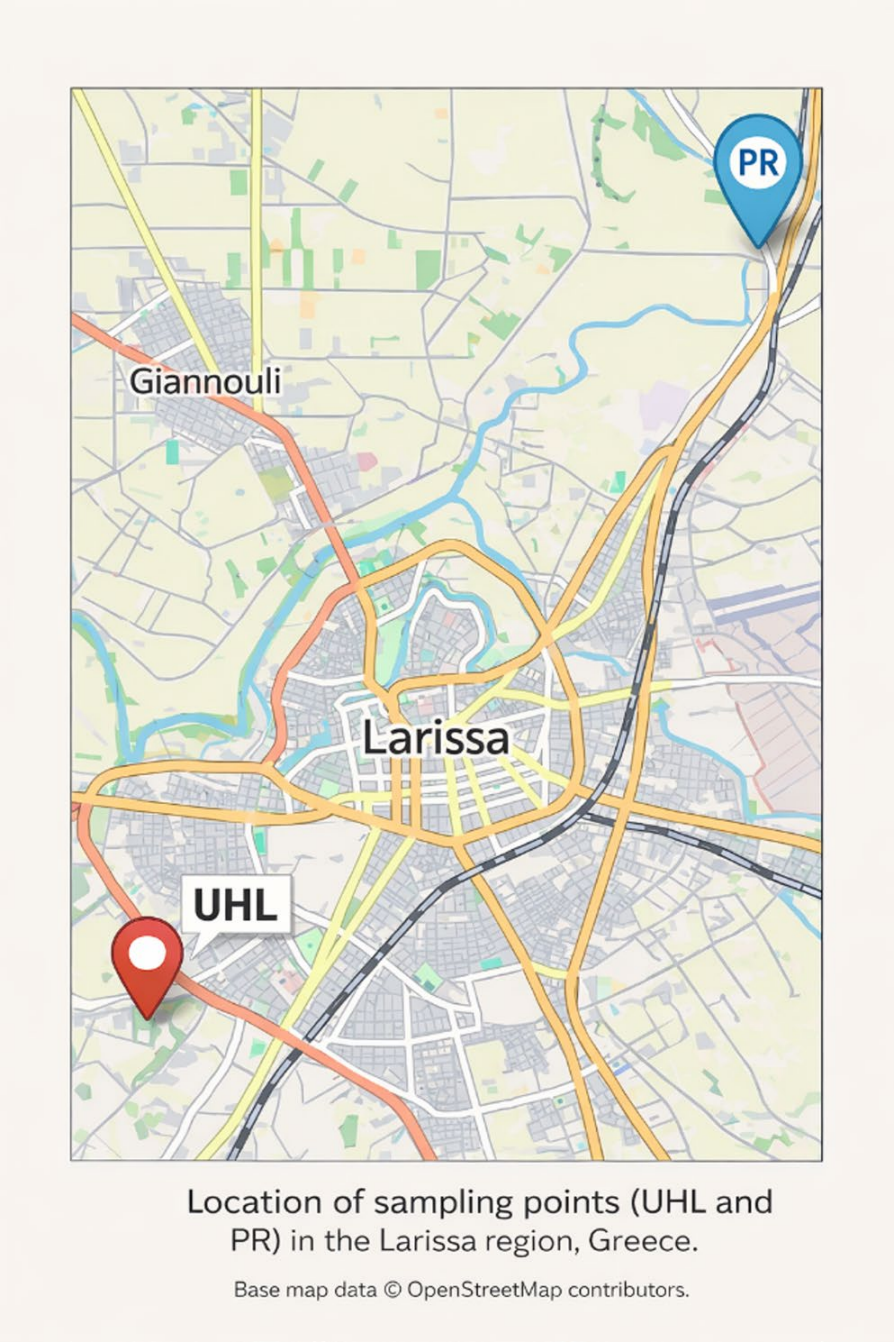
Geographic sampling points. With the red point is the location where sample of hospital wastewater collected (UHL) [39^o^ 36’ 38.79’’ N, 22^o^ 23’ 8.89’’ E] and with the blue point is where sample of river water collected (PR) [39^o^ 40’ 31.64’’ N, 22^o^ 26’ 50.44’’ E] in the city of Larissa, Greece. Map generated by the authors; base geographic features inspired by OpenStreetMap

Two water samples were collected: wastewater (effluent) from the University Hospital of Larissa (UHL) [39o 36’ 38.79’’ N, 22o 23’ 8.89’’ E], and river water from Pineios River at a sampling point after wastewater treatment plant (WWTP) [39o 40’ 31.64’’ N, 22o 26’ 50.44’’ E]. More specifically, the sampling points are shown in Fig. 1. The samples were collected in February 2025. Collection of samples as performed, as described previously (Gagaletsios et al., 2025).

### Bacterial isolation and identification

Our study focused on finding gram-negative bacteria that may be present in these samples. Directly after the collection, samples were vortexed and enriched in Luria-Bertani (LB) broth, under aerobic conditions at 37 °C overnight. Subsequently, serial dilutions of each culture were plated onto McConkey agar containing meropenem (4 mg/mL). Plates were incubated at 37 °C overnight. Next day, individual colonies of bacteria were selected based on phenotypic characteristics and sub-cultured on the same medium, to separate the different species from each other.

For species identification, MALDI-TOF mass spectrometry was used, which analyzes the molecular composition of bacterial proteins (Singhal et al. 2015).

### Antibiotic susceptibility testing

Antibiotic Susceptibility testing of the isolates (*n* = 54) was performed using the broth microdilution method to determine the minimum inhibitory concentrations (MICs) (EUCAST 2003). The results were evaluated according to EUCAST breakpoints (https://www.eucast.org/fileadmin/eucast/pdf/breakpoints/v_15.0_Breakpoint_Tables.pdf).

### Whole-genome sequencing and analysis

To gain a comprehensive overview of the isolates population recovered from both hospital wastewater and river water, whole-genome sequencing (WGS) was performed on a subset of selected isolates (*n* = 27), based on species identification and susceptibility profiles. Bacterial DNA was extracted using magnetic beads with the Genomic DNA Purification Kit (Thermo Scientific™, Waltham, Massachusetts, United States). Sequencing was carried out using the PacBio long-read platform (Bitar et al., 2023), followed by *de novo* assembly from PacBio-generated HiFi reads leveraging their long length and high accuracy, using the Microbial Assembly pipeline offered by the SMRT Link v10.0 software.

Multilocus Sequencing Typing (MLST) of the strains were detected with MLST 2.0 (Larsen et al., 2012) (accessed on 10 September 2025), selecting the correct MLST configuration, 5x as minimum depth for an allele and assembled genome/contigs as type of data input. Antimicrobial resistance genes were detected with ResFinder 4.6 (Bortolaia et al. 2020) (https://genepi.food.dtu.dk/resfinder, accessed on 10 September 2025), applying thresholds of ≥ 90% for minimum identity and ≥ 60% for minimum coverage. Furthermore, plasmid replicons were identified using PlasmidFinder 2.1 (Carattoli et al. 2014) (https://cge.food.dtu.dk/services/PlasmidFinder/, accessed on 10 September 2025) with thresholds set at ≥ 95% identity and ≥ 60% coverage. Comparative plasmid alignment was done using BLAST.

Ring Image Generator (BRIG) (Alikhan et al. 2011).

Moreover, the SNPs between the genomes of same STs were detected using snippy multicommand (snippy-base application v4.5.0) (Seeman 2015) which generates a core genome multiple alignment against the reference. The pipeline detects the variants and generates a single file for each isolate listing the different variations.

### Nucleotide sequence accession numbers

Whole-genome assemblies of isolates were uploaded in NCBI with the accession number PRJNA1419577.

## Results

### Bacterial identification

From wastewater sample, a total of 38 meropenem-resistant isolates were collected based on phenotypic characteristics. These isolates were identified as *Enterobacterales* (*n* = 30; *Enterobacter spp.* [*n* = 15], *Citrobacter spp.* [*n* = 9], *Klebsiella pneumoniae* [*n* = 4], and *Raoultella ornithinolytica* [*n* = 2]), Pseudomonadales (*n* = 7) and Aeromonadales (*n* = 1) (Table 1). On the other hand, from the river sample, 16 isolates were collected. These isolates were identified as Aeromonadales (*n* = 12), and *Enterobacterales* (*n* = 4; *Citrobacter freundii* [*n* = 2], *Acinetobacter sp*. [*n* = 1], and *K. pneumoniae* [*n* = 1]) (Table 1).

**Table 1:** Number of bacteria isolated at each sampling point, hospital wastewater and river water in Larissa, 2025

Isolates	Hospital wastewater	Pineios river	Total
*Acinetobacter baumannii.*	0	1	1
*Aeromonas caviae*	1	6	7
*Aeromonas diversa*	0	1	1
*Aeromonas sp.*	0	2	2
*Aeromonas veronii*	0	3	3
*Citrobacter amalonaticus*	1	0	1
*Citrobacter braakii*	2	0	2
*Citrobacter freundii*	5	2	7
*Citrobacter koseri*	1	0	1
*Enterobacter cloacae complex*	9	0	9
*Enterobacter kobei*	1	0	1
*Enterobacter roggenkampii*	5	0	5
*Klebsiella pneumoniae*	4	1	5
*Pseudomonas aeruginosa*	1	0	1
*Pseudomonas alcaligenes*	1	0	1
*Pseudomonas oleovorans*	3	0	3
*Pseudomonas shirazica*	1	0	1
*Pseudomonas sp.*	1	0	1
*Raoultella ornithinolytica*	2	0	2
Overall	**38**	**16**	**54**

### Antibiotic resistance

**Fig. 2:**
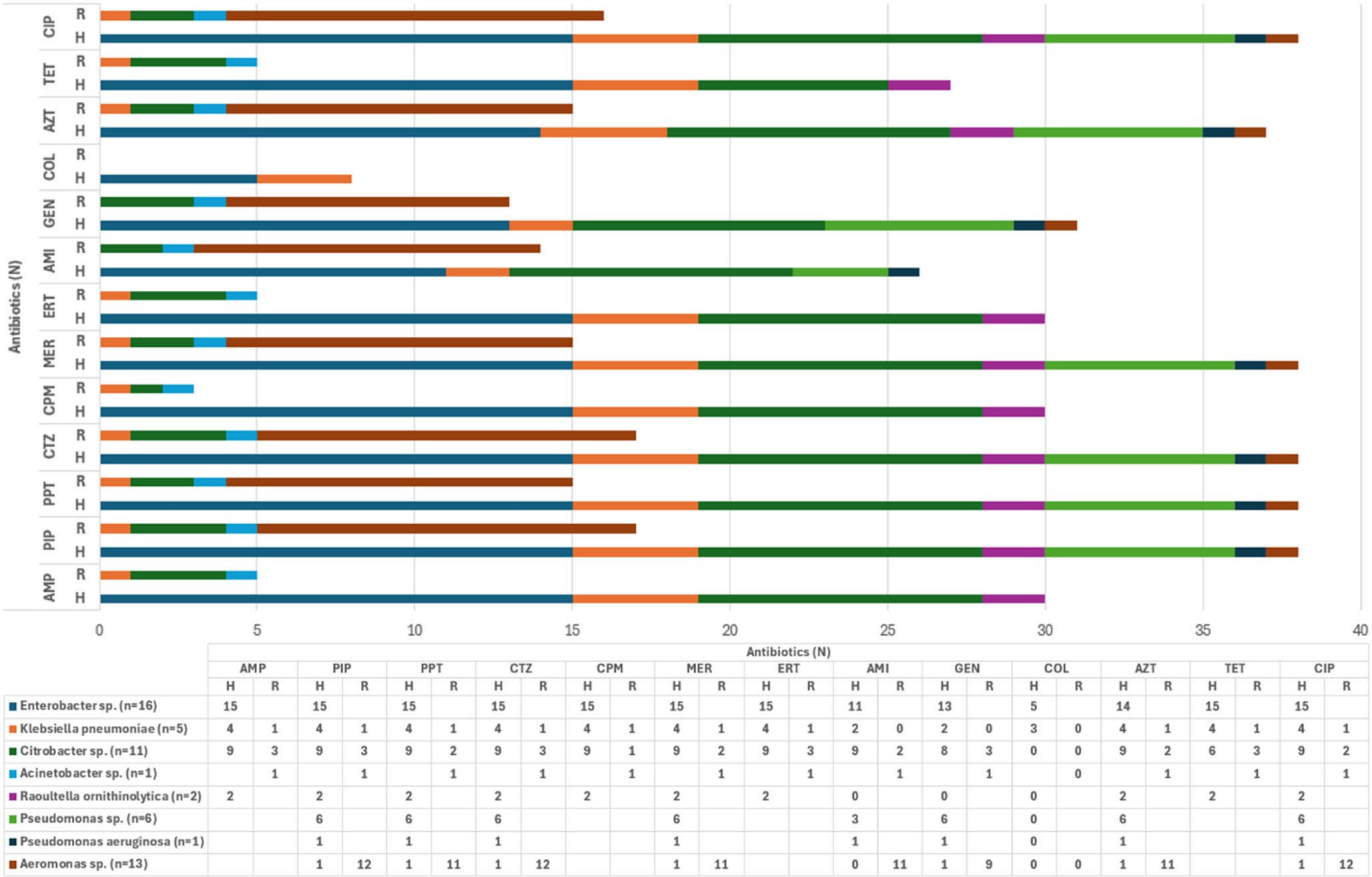
Resistance rate of gram-negative isolates (*H* Hospital wastewater; *R* River water) to commonly used antibiotics, 2025. *AMP* Ampicillin, *PIP* Piperacillin, *PPT* Piperacillin-Tazobactam, *CTZ* Ceftazidime, *CPM* Cefepime, *MER* Meropenem, *ERT *Etrapenem, *AMI* Amikacin, *GEN* Gentamicin, *COL* Colistin, *AZT* Aztreonam, *TET* Tetracycline, *CIP* Ciprofloxacin

Antibiotic susceptibility testing showed that all isolates (100%) from wastewater were resistant to ampicillin-sulbactam, piperacillin, piperacillin-tazobactam, ceftazidime, meropenem and ciprofloxacin, 33 isolates were resistant to aztreonam, 26 (68.4%) were resistant to trimethoprim-sulfamethoxazole, 23 (60.5%) were resistant to gentamicin, 13 (34.2%) were resistant to amikacin, and 10 (26.3%) were resistant to tigecycline, whereas only 5 (13.2%) isolates were resistant to colistin. Also, all river isolates (100%) were resistant to ampicillin-sulbactam, piperacillin, and ceftazidime, while 15 (93.8%) were resistant to piperacillin-tazobactam and meropenem, and 10 (62.5%) were resistant to aztreonam. Additionally, all isolates (100%) exhibited resistance to ciprofloxacin, 13 (81.3%) were resistant to gentamicin, 12 (75.0%) were resistant to amikacin, 10 (62.5%) were resistant to trimethoprim-sulfamethoxazole, while only 3 (18.8%) isolates were resistant to tigecycline. No river isolates were resistant to colistin (Fig. 2). Based on susceptibility profiles, all isolates were classified as MDR (multidrug resistance), as each isolate exhibited resistance to at least one agent from more than three different antibiotic classes (Magiorakos et al., 2012).

### WGS data analysis

Based on species identification and susceptibility profiles, 27 isolates (19 from wastewater and 8 from river-water) were selected to be further characterized by WGS. The data showed that *Aeromonas caviae* isolate belonged to ST820, isolates of *Citrobacter spp*. belonged to diverse STs (Table 2), while the 5 *E. roggenkampii* isolates were grouped to ST156 and the *E. kobei* isolate was ST910. Two out of three *K. pneumoniae* were ST307, whereas the third isolate was ST258. Among the Pseudomonadales, the *P. aeruginosa* isolate was assigned to ST357, the *P. shirazica* isolate was assigned to ST17, while the three remaining isolates belong to species that cannot be typed by MLST schemes. On the other hand, about environmental isolates, *Aeromonas* isolates were assigned to two distinct STs (ST422 [*n* = 2], and ST3590 [*n* = 2]), both *C. freundii* isolates belonged to ST167, and the *A. baumannii* was ST2. Finally, the *K. pneumoniae* was assigned to ST11. Among environmental isolates, STs, like *C. freundii* ST167, *A. baumannii* ST2 and *K. pneumoniae* ST11, which have been previously described from clinical isolates were found (Woodford et al. 2011).

**Table 2:** Characteristics of 27 bacterial strains, characterized by PacBio HiFi sequencing

Isolate	Strain	Origin	ST	Resistance genes	Plasmid replicons	AMR profile
LYM8H12	*A. caviae*	hospital wastewater	820	*bla*_*KPC−2*_, *bla*_VIM−1_, *bla*_OXA−10_, *bla*_TEM−1 A_, *aadA1*, *aadA11*, *aac(*6’*)-Ib3*, *dfrA14*	*IncP6*	PIP, PPT, CTZ, MER, GEN, AZT, CIP
LYM8H2	*C. amalonaticus*	hospital wastewater	758	*bla*_*VIM−1*_, *bla*_OXA−1_, *bla*_OXA−10_, *arr-3*,* aac(6’)-Ib-cr*,* aac(6’)-Il*,* aadA22*,* ant(2’’)-Ia*,* aph(3’)-Ia*,* aph(3’)-VIa*,* catB3*,* dfrA1*,* mph(B)*,* qnrA1*,* qnrS1*,* sul1*,* tet(B*)	IncHI2, IncHI2A, *IncC*, IncFIB(K), IncFIA(HI1), IncN	AMP, PIP, PPT, CTZ, CPM, MER, ERT, AMI, GEN, AZT, TET, CIP
LYM8H15	*C. freundii*	hospital wastewater	19	*bla*_*KPC−2*_, *bla*_NDM−1_, *bla*_OXA−1_, *bla*_OXA−10_, *bla*_TEM−1B_, *bla*_CMY−152_, *arr-3*,* aac(6’)-Ib-cr*,* aadA1*,* aadA16*,* catB3*,* dfrA27*,* mph(A)*,* rmtB*,* sul1*,* tet(G)*	IncFII(Cf), *IncFIB(pQil)*, *IncFII(K)*, IncR	AMP, PIP, PPT, CTZ, CPM, MER, ERT, AMI, GEN, AZT, TET, CIP
LYM4H12	*C. braakii*	hospital wastewater	769	*bla*_*KPC−2*_, *bla*_TEM−1 A_, *bla*_CMY−83_, *aac(3)-I*,* aac(6’)-Ib3*,* aadA1*,* qnrB68*,* sul1*	IncFIB(K), IncFIB(pHCM2), *IncP6*, IncX5	AMP, PIP, PPT, CTZ, CPM, MER, ERT, AMI, GEN, AZT, TET, CIP
LYM4H19	*C. braakii*	hospital wastewater	1412	*bla*_*KPC−2*_, *bla*_VIM−1_, *bla*_TEM−1 A_, *bla*_CMY−93_, *aac(6’)-Ib3*,* qnrB10*,* sul1*	IncC, IncFIB(pHCM2), *IncP6*, pKPC-CAV13, ColpVC	AMP, PIP, PPT, CTZ, CPM, MER, ERT, AMI, AZT, CIP
LYM4H18	*E. kobei*	hospital wastewater	910	*bla*_GES−7_, aac(6’)-Ib3, aadA1, *dfrA1*,* sul1*	IncFIB(K), IncP6, repB(R1701)	AMP, PIP, PPT, CTZ, CPM, MER, ERT, AMI, AZT, TET, CIP
LYM4H6	*E. roggenkampii*	hospital wastewater	771	*bla*_GES−5_, *bla*_OXA−10_, *aac(6’)-Ib3*,* aadA1b*,* aph(3’’)-Ib*,* aph(3’)-VI*,* aph(6)-Id*,* mph(E)*,* msr(E)*	IncFIB(pECLA), IncFII(pECLA), IncFII(Yp), IncP6, pKPC-CAV1321	AMP, PIP, PPT, CTZ, CPM, MER, ERT, AMI, GEN, AZT, TET, CIP
LYM4H10	*E. roggenkampii*	hospital wastewater	771	*bla*_GES−5_, *bla*_OXA−10_, *aac(6’)-Ib3*,* aadA1b*,* aph(3’’)-Ib*,* aph(3’)-VI*,* aph(6)-Id*,* mph(E)*,* msr(E)*	IncFIB(pECLA), IncFII(pECLA), IncFII(Yp), IncP6, pKPC-CAV1321, Col(CriePir75), Col440I	AMP, PIP, PPT, CTZ, CPM, MER, ERT, AMI, GEN, AZT, TET, CIP
LYM8H7	*E. roggenkampii*	hospital wastewater	771	*bla*_GES−5_, *bla*_OXA−10_, *aac(6’)-Ib3*,* aadA1b*,* aph(3’’)-Ib*,* aph(3’)-VI*,* aph(6)-Id*,* sul1*	IncFIB(pECLA), IncFII(pECLA), IncX5, IncP6, pKPC-CAV1321, Col(CriePir75), Col440I, ColpVC	AMP, PIP, PPT, CTZ, CPM, MER, ERT, AMI, GEN, AZT, TET, CIP
LYM8H9	*E. roggenkampii*	hospital wastewater	771	*bla*_GES−5_, *bla*_OXA−10_, *aac(6’)-Ib3*,* aadA1b*,* aph(3’’)-Ib*,* aph(3’)-VI*,* aph(6)-Id*,* sul1*	IncFIB(pECLA), IncFII(pECLA), IncX5, IncP6, pKPC-CAV1321, Col(CriePir75), Col440I, ColpVC	AMP, PIP, PPT, CTZ, CPM, MER, ERT, GEN, AZT, TET, CIP
LYM8H16	*E. roggenkampii*	hospital wastewater	771	*bla*_GES−5_, *bla*_OXA−10_, *aac(6’)-Ib3*,* aadA1b*,* aph(3’’)-Ib*,* aph(3’)-VI*,* aph(6)-Id*,	IncFIB(pECLA), IncFII(pECLA), IncX5, IncP6, pKPC-CAV1321, Col(CriePir75)	AMP, PIP, PPT, CTZ, CPM, MER, ERT, COL, AZT, TET, CIP
LYM8H19	*K. pneumoniae*	hospital wastewater	258	*bla*_*KPC−2*_, aac(6’)-Ib	*IncFIB(K)*, *IncFII(K)*, ColRNAI	AMP, PIP, PPT, CTZ, CPM, MER, ERT, AMI, GEN, COL, AZT, TET, CIP
LYM8H17	*K. pneumoniae*	hospital wastewater	307	*bla*_*KPC−2*_, *bla*_CTX−M−15_, *bla*_OXA−1_, *bla*_TEM−1B_, *aac(6’)-Ib-cr*,* aph(3’’)-Ib*,* aph(6)-Id*,* catB3*,* dfrA14*,* qnrB1*,* sul2*,* tet(A)*	IncFIB(K), *IncFII(K)*, *IncFIB(pQil)*, IncFIB(pNDM-Mar), IncHI1B(pNDM-MAR), Col(pHAD28)	AMP, PIP, PPT, CTZ, CPM, MER, ERT, COL, AZT, TET, CIP
LYM8H18	*K. pneumoniae*	hospital wastewater	307	*bla*_*KPC−2*_, *bla*_CTX−M−15_, *bla*_OXA−1_, *bla*_TEM−1B_, *aac(6’)-Ib-cr*,* aph(3’’)-Ib*,* aph(6)-Id*,* catB3*,* dfrA14*,* qnrB1*,* sul2*,* tet(A)*	IncFIB(K), *IncFII(K)*, *IncFIB(pQil)*, IncFIB(pNDM-Mar), IncHI1B(pNDM-MAR), Col(pHAD28)	AMP, PIP, PPT, CTZ, CPM, MER, ERT, COL, AZT, TET, CIP
LYM4H1	*P. aeruginosa*	hospital wastewater	357	*bla*_VIM−2_, *aac(6’)-Ib3*,* ant(2’’)-Ia*,* aph(3’’)-Ib*,* aph(3’)-IIb*,* aph(3’)-Ia*,* aph(6)-Id*,* catB7*,* sul1*	–	PIP, PPT, CTZ, MER, AMI, GEN, AZT, CIP
LYM4H7	*P. alcaligenes*	hospital wastewater	NA	*bla*_VIM−2_, *aadA7*,* ant(3’’)-Ii-aac(6’)-IId*,* aph(3’’)-Ib*,* aph(6)-Id*,* sul1*	–	AMP, PIP, PPT, CTZ, MER, AZT, CIP
LYM8H1	*P. oleovorans*	hospital wastewater	NA	*bla*_VIM−1_, *bla*_VIM−2_, *bla*_OXA−10_, *bla*_OXA−14_, *aac(6’)-Ib3*,* aadA11*,* aadA7*,* ant(2’’)-Ia*,* aph(3’’)-Ib*,* aph(3’)-Ia*,* sul1*	–	AMP, PIP, PPT, CTZ, MER, GEN, AZT, CIP
LYM4H8	*P. oleovorans*	hospital wastewater	NA	*bla*_VIM−1_, *bla*_VIM−2_, *bla*_OXA−10_, *bla*_OXA−14_, *aadA7*,* aadA11*,* ant(2’’)-Ia*,* aac(6’)-Il*,* sul1*	–	AMP, PIP, PPT, CTZ, MER, GEN, CIP
LYM8H4	*P. shirazica*	hospital wastewater	17	–	–	AMP, MER, AZT
LYM4H20	*R. ornithinolytica*	hospital wastewater	NA	*bla*_*KPC−2*_, *bla*_VIM−1_, *bla*_OXA−9_, *bla*_TEM−1 A_, *aac(6’)-Ib3*,* sul1*	IncFIA(HI1), *IncFIB(pQil)*, *IncFII(K)*	AMP, PIP, PPT, CTZ, CPM, MER, ERT, AZT, TET, CIP
LYMR17	*A. baumannii*	river water	2	*bla*_OXA−23_, *bla*_OXA−66_, *aph(6)-Id*,* aph(3’’)-Ib*,* aph(3’)-VIa*,* armA*,* catA1*,* msr(E)*,* mph(E)*,* sul1*,* tet(B)*	–	AMP, PPT, CTZ, CPM, MER, ERT, AMI, GEN, AZT, TET, CIP
LYMR4	*A. caviaeenteropelogenes*	river water	3590	*bla*_*KPC−2*_, *bla*_VEB−1_, *bla*_OXA−10_, *bla*_TEM−1B_, *arr-2*,* ant(2’’)-Ia*,* aadA1*,* aph(3’’)-Ib*,* aph(6)-Id*,* cmlA1*,* rmtB*,* sul1*,* sul2*,* tet(G)*	*IncC*	PPT, CTZ, MER, AMI, GEN, AZT, CIP
LYMR10	*A. caviae*	river water	3590	*bla*_*KPC−2*_, *bla*_VEB−1_, *bla*_OXA−10_, *bla*_TEM−1B_, *arr-2*,* ant(2’’)-Ia*,* aadA1*,* aph(3’’)-Ib*,* aph(6)-Id*,* cmlA1*,* rmtB*,* sul1*,* sul2*,* tet(G)*	*IncC*	PPT, CTZ, MER, AMI, GEN, AZT, CIP
LYMR8	*A. veronii*	river water	422	*bla*_*VIM−4*_, *bla*_OXA−10_, *aac(6’)-Ib-Hangzhou*,* aph(3’’)-Ib*,* aph(3’)-VI*,* aph(6)-Id*,* tet(E)*	*IncP6*	PPT, CTZ, MER, AMI, GEN, AZT, CIP
LYMR16	*A. veronii*	river water	422	*bla*_*VIM−4*_, *bla*_OXA−10_, *aac(6’)-Ib-Hangzhou*,* aph(3’’)-Ib*,* aph(3’)-VI*,* aph(6)-Id*,* tet(E)*	*IncP6*	PPT, CTZ, MER, AMI, GEN, AZT, CIP
LYMR12	*C. freundii*	river water	167	*bla*_*KPC−2*_, *bla*_OXA−9_, *bla*_TEM−1 A_, *bla*_TEM−1B_, *bla*_CMY−152_, *aac(3)-IId*,* aac(6’)−29a*,* aac(6’)−29b*,* aadA2*,* dfrA12*,* mph(A)*,* qnrS2*,* sul1*	*IncFIB(pQil)*, *IncFII(K)*, IncHI1A(NDM-CIT), IncHI1B(pNDM-CIT), IncR, IncU	AMP, PPT, CTZ, MER, ERT, AMI, GEN, AZT, TET, CIP
LYMR14	*C. freundii*	river water	167	*bla*_*KPC−2*_, *bla*_OXA−9_, *bla*_TEM−1 A_, *bla*_TEM−1B_, *bla*_CMY−152_, *aac(3)-IId*,* aac(6’)−29a*,* aac(6’)−29b*,* aadA2*,* dfrA12*,* mph(A)*,* qnrS2*,* sul1*	*IncFIB(pQil)*, IncFII(K), IncHI1A(NDM-CIT), IncHI1B(pNDM-CIT), IncR, IncU	AMP, PPT, CTZ, MER, ERT, AMI, GEN, AZT, TET, CIP
LYMR1	*K. pneumoniae*	river water	11	*bla*_*NDM−1*_, *bla*_OXA−10_, *aac(6’)-Ib*,* aac(6’)-Ib3*,* aadA1*,* sul1*,* tet(A)*	IncFIB(K), IncFII(K), *IncFII(pKPX1)*, repB, *repB(R1701)*, Col440I	AMP, PPT, CTZ, CPM, MER, ERT, AZT, TET, CIP

Plasmid replicons associated with carbapenemase-encoding genes have been underlined

Analysis of WGS data using ResFinder confirmed that all isolates (except *P. shirazica* LYM8H4) recovered from wastewater sample carried carbapenemase-encoding genes. The *bla*_GES_ gene, *bla*_GES−5_ and *bla*_GES−7_ alleles, that were identified among *Enterobacter* isolates was the most common carbapenemase-encoding gene (Table 2). The *bla*_KPC−2_ gene was found in 4 isolates, while 4 other isolates were positive for the presence of two carbapenemase-encoding genes (*bla*_KPC−2_ and *bla*_VIM−1_ [*n* = 3], *bla*_KPC−2_ and *bla*_NDM−1_ [*n* = 1]). Three isolates were positive for *bla*_VIM_ alleles (*bla*_VIM−2_ [*n* = 2], *bla*_VIM−1_ [*n* = 1]), whereas the two *P. pseudoalcaligenes* isolates coproduced VIM-1 and VIM-2 metallo-β-lactamases. Also, carbapenemase-encoding genes were identified in all isolates of environmental origin. Four isolates (two *Aeromonas* and two *Citrobacter*) were positive for *bla*_KPC−2_, two *A. veronni* isolates were positive for *bla*_VIM−4_, and the ST11 *K. pneumoniae* isolate was positive for *bla*_NDM−1_. The *A. baumannii* isolate carried the *bla*_OXA−23_ carbapenemase-encoding gene. Furthermore, almost all isolates carried additional genes conferring resistance to aminoglycosides (*aac(6’)-Ib*,* aac(6’)-Ib3*, *aac(6’)-Ib-cr*,* aac(6’)-Il*, *aac(3)-IId*,* aac(6’)−29a*,* aac(6’)−29b*, *aadA1*, *aadA2*, *aadA11*,* aadA7*, *aph(3’’)-Ib*,* aph(3’)-Ia*), chloramphenicol (*catB3*, *cmlA1*), macrolides (*mph(A)*, *mph(B)*, *mph(E)*, *msr(E)*), quinolones (*qnrB1*, *qnrB10*, *qnrB68*, *qnrS2*), sulfonamides (*sul1*, *sul2*), tetracyclines (*tet(A)*, *tet(B*), *tet(E)*, *tet(G)*) and trimethoprim (*dfrA1*, *dfrA12*, *dfrA14*) (Table 2).

Analysis of WGS data using PlasmidFinder identified a huge variety of plasmid replicons among the isolates studied, with IncF-type replicons being the most common replicons (Table 2). Additionally, our analysis showed the association of *bla*_KPC−2_ with replicons IncFIB(pQil)-IncFII(K) (*n* = 6), IncP6 (*n* = 3), IncC (*n* = 3) and IncFIB(K)-IncFII(K) (*n* = 1). Also, we observed the association of *bla*_NDM−1_ with replicons IncFII(pKPX1)-repB(R1701) (*n* = 1) and IncR (*n* = 1), of *bla*_VIM−4_ with IncP6 (*n* = 2), and of *bla*_VIM−1_ with IncC (*n* = 1). Of note was that most isolates carried additional plasmids that couldn’t be typed by PlasmidFinder. However, almost all *Pseudomonas* and *Acinetobacter* carried no plasmids. In the two *P. oleovorans* isolates, megaplasmids that carried *bla*_VIM−1_ gene were found.

Furthermore, BlastN comparison showed that *bla*_KPC−2_-carring plasmids, typed to IncFIB(pQil)-IncFII(K) group, that were characterized from environmental isolates were significantly different from the respective plasmids of clinical origin, and the archetypal IncFIIK2 KPC-encoding plasmid pKpQIL(GenBank accession no. NC_014016), originally described in the ST258 K. pneumoniae Kpn557 isolated in Israel in 2006, and then reported worldwide (Figure S1) (Leavitt et al. 2010).

The two megaplasmids, carrying the *bla*_VIM−1_ gene, identified in two *P. pseudoalcaligenes* isolates exhibited extensive similarity to each other (63% coverage 97.52% identity), and to plasmids, like H15 (GenBank accession no. CP093017), DN1 (GenBank accession no. CP018048), 25,181 (GenBank accession no. CP173153), and pKF715A (GenBank accession no. AP015030), previously described from *Pseudomonas* isolates (Figure S1). However, the later plasmids were negative for the presence of the *bla*_VIM−1_ gene. In addition, the sequences surrounding the *bla*_VIM−1_ gene showed similarity to a transposition module previously characterized in the VIM-2-producing *P. aeruginosa* isolate Pae-32301cz (GenBank accession no. KY860568) from Czechia (Papagiannitsis et al. 2017).

Finally, as it can be observed from Table 2, isolates belonging to the same ST, like ST771 *E. roggenkampii* and ST307 *K. pneumoniae* from wastewater, and ST3590 *A. caviae*, ST422 *A. veronii* and ST167 *C. freundii* exhibited identical genomic features. SNP analysis confirmed that ST307 *K. pneumoniae*, ST3590 *A. caviae*, ST422 *A. veronii*, ST167 *C. freundii* were identical exhibiting less than 10 SNPs, while the ST771 *E. roggenkampii* were closely related differing by 78 to 150 SNPs. Finally, the two *P. oleovorans* isolates belonged to different clones, since they exhibited 37,820 SNPs.

## Discussion

In the current study, we examined whether potentially pathogenic bacteria from hospital wastewater can enter the environment, even after the wastewater has been treated in WWTP. Our results showed the presence of various MDR bacteria in the wastewater sample consistent with the previous findings from our lab (Gagaletsios et al., 2025). KPC-2-prducing *K. pneumoniae* isolates belonging to STs 258 and 307 have been previously described from clinical samples (Gagaletsios et al. 2025b; Chatzidimitriou et al. 2025). ST258 *K. pneumoniae* is the most common ST associated with the spread of *bla*_KPC_ gene (Afolayan et al., 2023). ST307 *K. pneumoniae* isolates have been characterized as an emerging high-risk clone associated with the dissemination of carbapenemases and *bla*_CTX−M−15_ (Chatzidimitriou et al. 2025).

Additionally, our results showed that the sample from the river contained pathogenic bacteria as well as bacteria containing genes conferring resistance to significant antimicrobial agents. Notably, *C. freundii* ST167, *A. baumannii* ST2 and *K. pneumoniae* ST11 have been previously described from clinical isolates (Mboowa et al. 2025; Galani et al. 2020; Woodford et al. 2011). ST11, which belongs to clonal complex (CC258) (Hu et al. 2025), has been described with different carbapenemase-encoding genes (*bla*_NDM_, *bla*_KPC_) (Afolayan et al., 2023). Although common bacterial species were found in both clinical and environmental samples, no similar STs and mobile elements were found. Given this substantial genetic heterogeneity, a direct paired influent–effluent–type comparison is not appropriate to interpret the lack of correlation as supporting the absence of direct contamination. Even in KPC-2-producing isolates with IncFIB(pQil)-IncFII(K) replicons, *bla*_KPC−2_-carrying plasmids characterized from environmental sample differed from the respective plasmids of clinical origin. Furthermore, a huge variety of plasmid replicons associated with resistance genes was found among the studied isolates of both origin (Table 2). This finding highlights the important role of mobile genetic elements in the spread of resistance genes and the evolution of MDR bacteria.

In our study, we cannot hypothesize the origin of MDR bacteria in the river sample. However, previous studies have reported that industrial and municipal wastewater, which are also rich in bacteria, discharges into WWTP (Boesten et al. 2011). However, a significant limitation of the current study is that sampling was conducted at a single time point (February 2025). The presentation of seasonal data could be significantly diverse, since microbial communities fluctuate significantly with seasons and rainfall. At the same time other studies reported enrichment of some resistance genes in the effluent (Alexander et al. 2015; Amador et al. 2015; Bengtsson-Palme et al. 2016; Pazda et al. 2019). Alexander et al. (2015) showed that WWTPs are not designed to remove ARGs. They observed the increase in the abundance of some ARGs in the bacterial population after conventional wastewater treatment. Thus, our findings highlight the need for measures to minimize this transmission.

## Conclusions

In conclusion, our findings confirm that hospital wastewater represents a significant source of multidrug-resistant bacteria, which persist in the environment post WWTP treatment. The presence of carbapenemase-producing isolates in river water underscores the need for advanced wastewater treatment methods and active surveillance to limit the transmission of environmental antimicrobial resistance.

## Supplementary Information

Below is the link to the electronic supplementary material.

Supplementary Material 1

Supplementary Material 2

## Data Availability

Whole-genome assemblies of isolates were uploaded in NCBI with the accession number PRJNA1419577.
