# Supplementary material for: Comparison of microbial profiles between hospital wastewater and river water
Source: Mol Genet Genomics. 2026 Jun 29;301(1):150. doi: 10.1007/s00438-026-02478-0 (PMC13315120; doi:10.1007/s00438-026-02478-0)
Supplement: Supplementary file 1 — Supplementary Material 1 [file 438_2026_2478_MOESM1_ESM.pptx]

## Slide 1
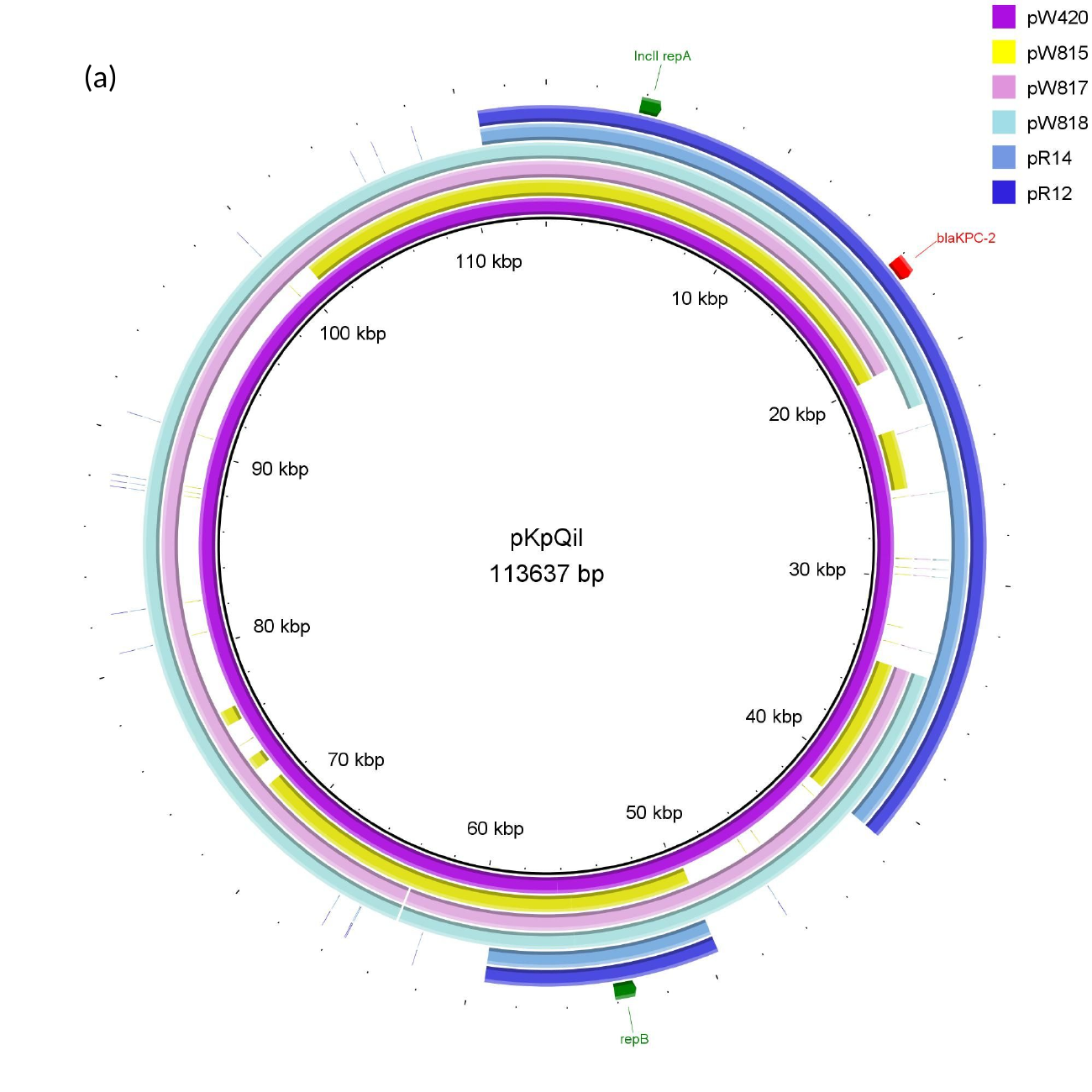

(a)

## Slide 2
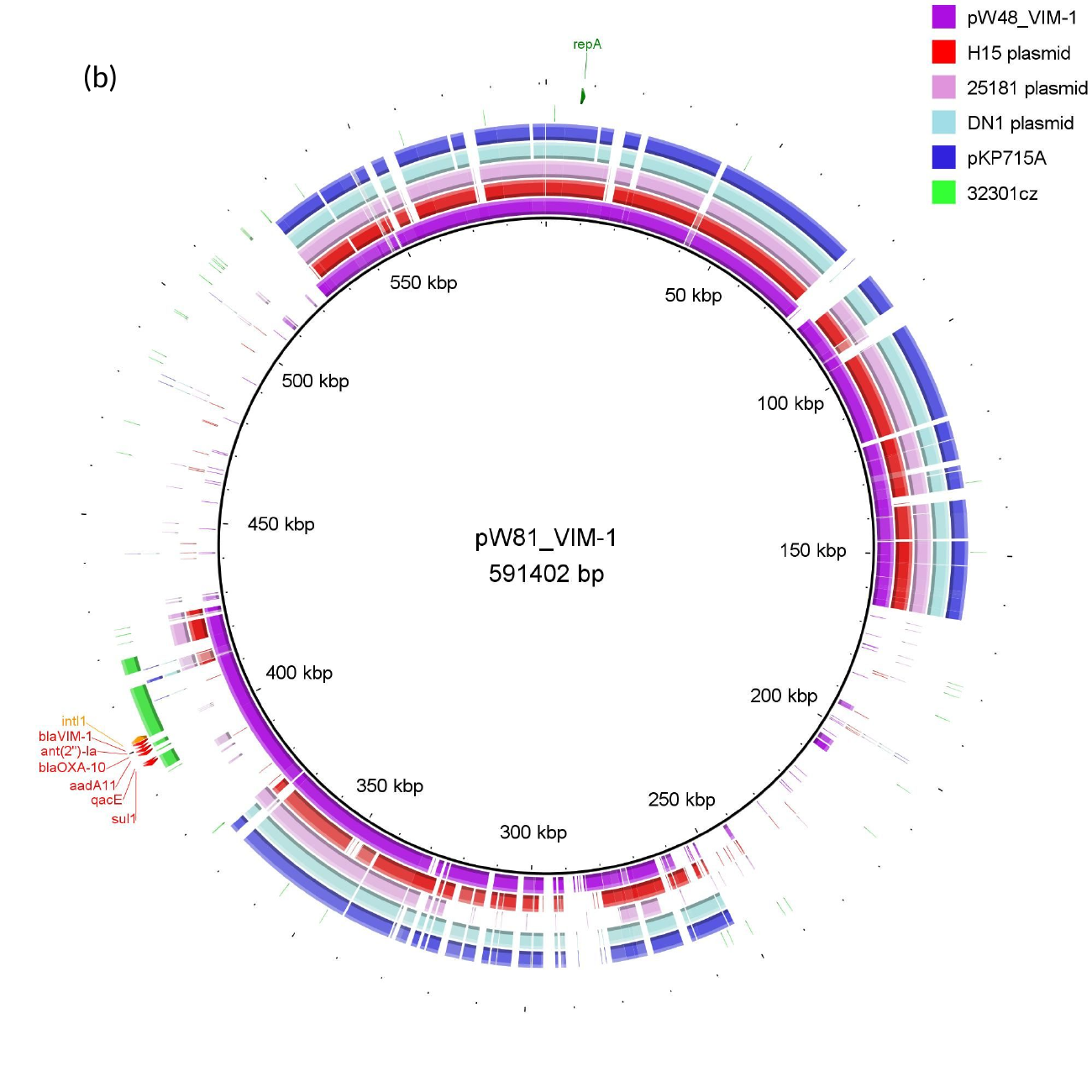

(b)

## Slide 3
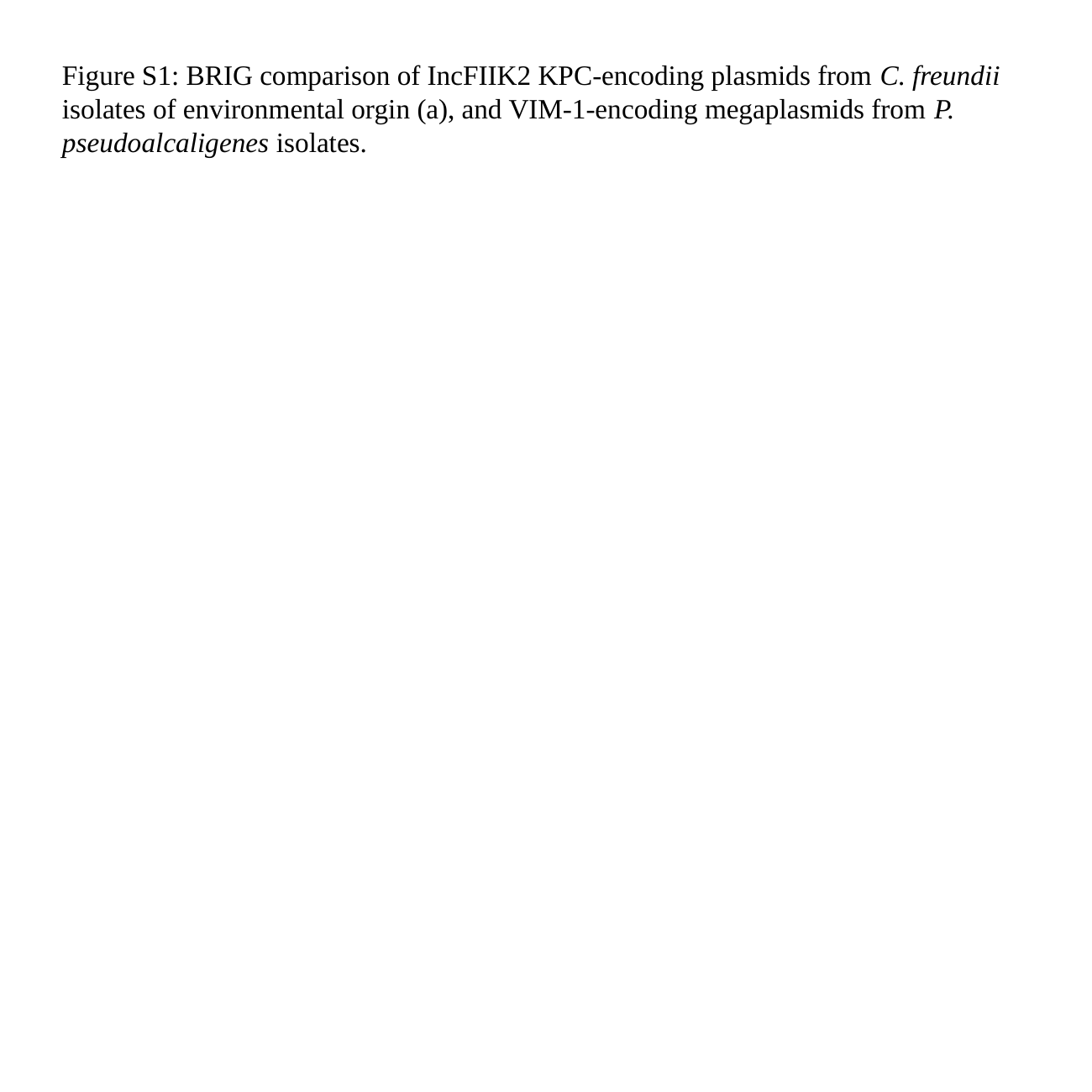

Figure S1: BRIG comparison of IncFIIK2 KPC-encoding plasmids from C. freundii isolates of environmental orgin (a), and VIM-1-encoding megaplasmids from P. pseudoalcaligenes isolates.
